# Recovering full-length viral genomes from metagenomes

**DOI:** 10.3389/fmicb.2015.01069

**Published:** 2015-10-01

**Authors:** Saskia L. Smits, Rogier Bodewes, Aritz Ruiz-González, Wolfgang Baumgärtner, Marion P. Koopmans, Albert D. M. E. Osterhaus, Anita C. Schürch

**Affiliations:** ^1^Department of Viroscience, Erasmus Medical CenterRotterdam, Netherlands; ^2^Department of Zoology and Animal Cell Biology, University of the Basque Country (UPV/EHU)Vitoria-Gasteiz, Spain; ^3^Systematics, Biogeography and Population Dynamics Research Group, Lascaray Research Center, University of the Basque Country (UPV/EHU)Vitoria-Gasteiz, Spain; ^4^Conservation Genetics Laboratory, National Institute for Environmental Protection and ResearchBologna, Italy; ^5^Department of Pathology, University of Veterinary Medicine HannoverHannover, Germany; ^6^Centre for Infectious Diseases Research, Diagnostics and Screening, National Institute for Public Health and the EnvironmentBilthoven, Netherlands; ^7^Center for Infection Medicine and Zoonoses ResearchHannover, Germany

**Keywords:** metagenomics, viruses, virus discovery, assembly, k-mer analysis, coverage analysis, motif discovery, zoonotic pathogens

## Abstract

Infectious disease metagenomics is driven by the question: “what is causing the disease?” in contrast to classical metagenome studies which are guided by “what is out there?” In case of a novel virus, a first step to eventually establishing etiology can be to recover a full-length viral genome from a metagenomic sample. However, retrieval of a full-length genome of a divergent virus is technically challenging and can be time-consuming and costly. Here we discuss different assembly and fragment linkage strategies such as iterative assembly, motif searches, k-mer frequency profiling, coverage profile binning, and other strategies used to recover genomes of potential viral pathogens in a timely and cost-effective manner.

## Introduction

Infectious viral diseases, both emerging, and re-emerging, pose a continuous health threat and disease burden to humans. In recent years, we have seen an increasing number of emerging virus disease outbreaks in human and animals alike with substantial health and economic impact. They are most likely related to accelerating environmental and anthropogenic changes, such as increased mobility and demographic changes, which alter the rate and nature of contact between animal and human populations. The influenza A viruses are probably the most notorious viruses which have shown their potential for repeated cross species transmission and pandemic potential (Claas et al., 1998; Koopmans, 2013; de Graaf and Fouchier, 2014; Bodewes et al., 2015). Schmallenberg virus caused an outbreak in ruminants with major impact on international trade of susceptible animals and animal products such as semen and embryos with more than 15 countries imposing restrictions on imports of live cattle from the European Union (EU) (Beer et al., 2013). Lately, Middle East Respiratory Syndrome (MERS) coronavirus was causing renewed concern as it spread from the Middle East to the Republic of Korea with 186 confirmed human cases, including 36 deaths in July 2015 (http://www.who.int/csr/don/21-july-2015-mers-korea/en/). Ebola virus has fruit bats (*Pteripodidae*) as natural hosts and the current epidemic is thought to be introduced into the human population by zoonotic transmission (Marí Saíz et al., 2014). Many of the most important human pathogens are either zoonotic or originated as zoonoses before adapting to humans (Taylor et al., 2001; Kuiken et al., 2005; Woolhouse and Gowtage-Sequeria, 2005; Cutler, 2010; Morse et al., 2012) and humanity is continuously being exposed to novel animal pathogens.

Breakthroughs in the field of metagenomics have had far-reaching effects on the identification and characterization of newly emerging viral pathogens (Fauci and Morens, 2012). Virus discovery metagenomics assays rely on sequence-independent amplification of nucleic acids from clinical samples, in combination with next-generation sequencing platforms and bioinformatics tools for sequence analysis. They are relatively simple and fast, and allow detection of hundreds of viruses simultaneously and unknown viruses even if they are highly divergent from those that are already described (Rosario and Breitbart, 2011; Miller et al., 2013; Smits and Osterhaus, 2013). If the new viral genome shows considerable similarity to previously characterized virus genomes present in public databases, the identification of a new virus and its genomic characterization can be finalized in a matter of days and a fraction of the costs compared to a few years ago. This is of utmost importance for timely disease outbreak management. Here, the guiding questions are: Is the group of diseased persons normal for the time of year and/or geographic area? If so, which pathogen(s) is causing the disease? Who gets infected? How do people get infected? What is the source of infection? What are transmission routes? How can infection be prevented, treated and/or contained? The fast discovery of a partial or full-length viral genome can also serve as basis for development of specific molecular diagnostic assays to confirm suspect cases and for development of vaccines and antivirals. This was exemplified after the discoveries of Schmallenberg virus and MERS Coronavirus, where molecular diagnostic protocols were made available within a matter of days after the discovery of the pathogen (Beer et al., 2013; Pollack et al., 2013).

**Figure 1:**
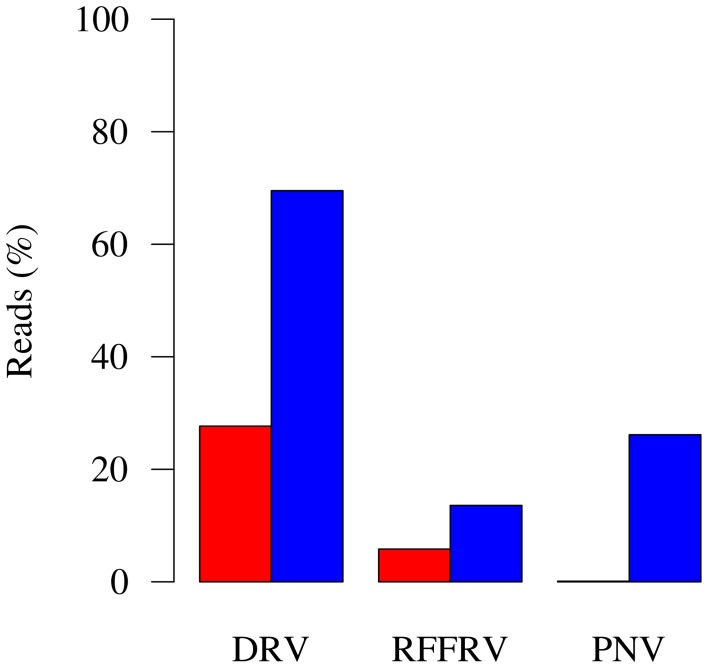
**Metagenome composition before and after genome finishing**. Red bars: Percentage of reads of the metagenome assigned to target genome after similarity searches and before genome finishing. Blue bars: Percentage of reads of the metagenome assigned to target genome after genome finishing strategies were applied to the target genome. DRV, dolphin rhabdovirus; RFFRV, red fox fecal rhabdovirus; PNV, python nidovirus.

**Figure 2:**
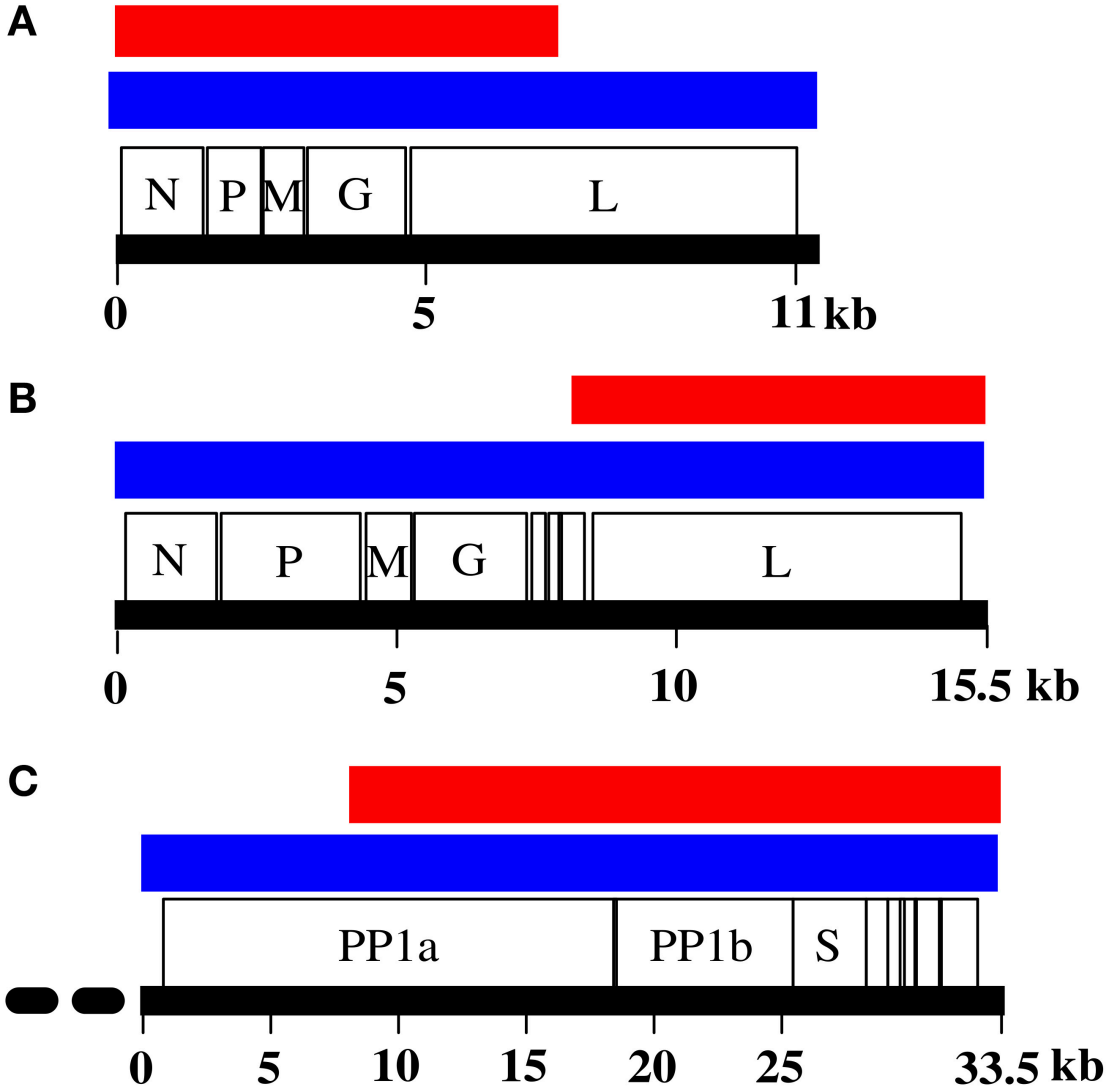
**Genome completeness before and after genome finishing**. **(A)** Genome of dolphin rhabdovirus (DRV). **(B)** Genome of red fox fecal rhabdovirus (RFFRV). **(C)** Genome of python nidovirus (PNV). Indicated is contig size after initial genome assembly (red) and after genome finishing (blue). Striped line at the 5′ end of PNV indicates putative unresolved 5′ end.

Despite this promise, however, most new discoveries made through metagenomics in fact are viruses that belong to already known virus families as current data analysis strategies rely mostly on **similarity searches** against annotated sequences in public databases (Woyke et al., 2006; Chew and Holmes, 2009; Schmieder and Edwards, 2012; Garcia-Garcerà et al., 2013; Prachayangprecha et al., 2014; Schürch et al., 2014). Significant problems in characterization of full-length viral genomes from metagenomic datasets are encountered when dealing with a highly divergent new virus with no closely related genomes in public databases. Additional time-consuming experimental approaches are required to obtain full-length genome sequences (Van Leeuwen et al., 2010; Siegers et al., 2014). By optimally mining the metagenomic content, for example through effective assembly, k-mer frequency profiling, motif search, coverage profile binning, or other fragment linkage strategies, the likelihood, and speed of finding viral reads and the level of viral genome completeness can be increased. This also increases the number of reads for which a source can be assigned in metagenomes. An example was described by us recently (Smits et al., 2014) and showed that, using BLAST searches, 27.67, 5.82, and 0.11% of all reads were identified as being from the viral target genome (Figure 1), whereas after viral **genome finishing**, 69.52, 13.58, and 26.14% reads were tagged as belonging to the viral genome (Figure 1). At the same time, genome completeness increased from 7291, 7682, and 24,734 bases in the initial fragments, to full-length or nearly full-length genomes of 11, 15.5, and 33 kb (Figure 2). With the *in silico* methods described here, the need for laboratory follow-up can be minimized, thereby providing the necessary information in a timely, efficient, and more cost-effective manner.

KEY CONCEPT 1 **Similarity searches**

Similarity searches in metagenomics, also referred to as homology searches, refers to searching of sequences databases for matching sequences, often with BLAST (Altschul, 1997), in order to assign a taxonomy to a query sequence.

KEY CONCEPT 2 **Genome finishing**

After metagenome assembly, genomes of individual organisms are often incomplete and shattered into different contigs. While in case of known viral genomes, the genomes can be resolved by mapping to a reference genome, in novel, and divergent viral genomes it is often necessary to link contigs first. Further steps to retrieve a full-length genome involve confirmation of linkage, augmenting assemblies, determination of the order of the contigs and closing gaps between them.

**Figure 3:**
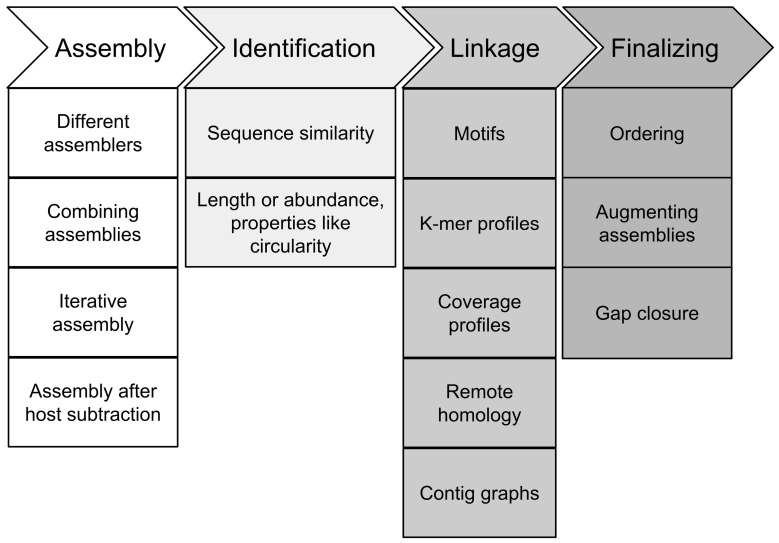
**Steps in recovery of full-length viral genomes from metagenomes**. For description see article text.

Recovery of full-length genomes of novel viruses from metagenome data consists of four different steps: First, the assembly of the reads into long fragments, second, assignment of at least one contig (seed) as originating from the target virus, third, the linkage of other fragments to the seed contig to receive a draft genome and fourth, gap closing and finalizing of the draft genome to receive a full-length genome (Figure 3).

## Assembly

A metagenome dataset consists not only of reads of the (novel viral) target genome but also of all other genetic material present in the sequenced sample, at varying concentrations and sequencing depth. If the viral load in the sample is sufficient to produce overlapping sequencing reads, it is in theory possible to create longer, contiguous sequences from the viral genome. A first step in retrieval of a viral genome from a metagenome is therefore to assemble all reads, followed by identification of (part of) the target genome. Unfortunately, only very few assemblers were developed with virus discovery metagenomics in mind. A specific challenge here is the much greater sequence diversity in viruses compared with host or bacterial genomes. In contrast to assembly in general metagenomics where assembly of all sequencing reads is necessary to shed light into population and functions within the metagenomic community (Howe and Chain, 2015), only assembly of a single or few targets (namely the virus in question) but to a high level of completeness is pursued. These targets might have highly uneven coverage and can be very dissimilar to sequences of known pathogens which make reference-guided assembly impossible.

A comparison of the performance of standard, whole-genome shotgun DNA sequence assemblers in viral metagenomes has been published recently (Vázquez-Castellanos et al., 2014). None of the tested assemblers achieved convincing results. Whole-genome assemblers are known to perform poorly on metagenomes because they assume even coverage (Pop, 2009; Laserson et al., 2011; Peng et al., 2011; Lai et al., 2012; Namiki et al., 2012; Scholz et al., 2012). An assembler that was designed to work with uneven depth encountered in metagenomes is IDBA-UD (Peng et al., 2011) and it has been tested for viral genome assembly in simulated viral metagenomes with good results (Aguirre de Cárcer et al., 2014).

If the genome sequence of the host is known and the target virus not endogenous, the reads generated from the host can be subtracted prior to assembly by mapping which can increase virus contig lengths (Daly et al., 2015).

Other successful approaches in assembly of viral genomes from mixed samples are strategies that involve **iterative assembly**. The genome of Bas-Congo virus that was associated with an outbreak of human cases of acute hemorrhagic fever in the Democratic Republic of Congo in 2009 (Grard et al., 2012), was assembled with software (PRICE) that applies iterative rounds of contig extension on paired-end reads (Ruby et al., 2013). This targeted way of assembly is depending on a seed sequence, such as a read or contig that was initially identified by sequence similarity to a known virus. The seed is then extended by establishing local assemblies at both ends with the remaining reads which are merged to form a new contig. The target genome grows with each extension step. PRICE has been applied to a number of metagenome datasets, and aided, among others, in the discovery of a frequent contaminant in spin columns (Naccache et al., 2013), the identification of novel rhabdoviruses and bunyaviruses in mosquitos (Coffey et al., 2014), and the assembly of a novel nidovirus from a ball python (Stenglein et al., 2014). A similar, iterative assembly approach is applied by IVA (Iterative Virus Assembler, Hunt et al., 2015), developed for RNA virus genomes with uneven sequencing depth. Extension steps are carried out more conservatively than with PRICE, leading to more accurate assemblies of HIV and Influenza sequencing samples. The usefulness of IVA for metagenome and virus discovery data has yet to be shown.

KEY CONCEPT 3 **Iterative assembly**

Sequence assembly consists of searches of overlaps, alignment, and merging of sequences. Computational limitations however prohibit most assemblers to perform exhaustive overlap searches. In iterative assembly, the resulting contiguous sequences (contigs) and singletons of the initial assembly are subjected to assembly by the same or a different assembly algorithm. This process is repeated until no new contigs can be found. Iterative assembly disregards coverage information and is therefore well-suited for metagenomics samples where coverage biases might exist.

Both assemblers were designed for paired-end short read data. To achieve the assembly of long 454 metagenome reads, an algorithm was developed that sequentially uses an overlap-layout-consensus (OLC) assembler (Newbler, Roche 454) followed by several rounds of greedy assembly (CAP3, Huang and Madan, 1999) until convergence (Schürch et al., 2014). Application of this algorithm to a data set derived from a cell culture supernatant containing a virus isolated from tissue of a dead white-beaked dolphin (*Lagenorhynchus albirostris*) lead to long contigs of a novel dolphin rhabdovirus that was closely related to fish rhabdoviruses of the genera *Perhabdovirus* and *Vesiculovirus* (Siegers et al., 2014). These contigs were combined with fragments derived by other assembly methods to retrieve a full viral genome (Smits et al., 2014). Such a sequential assembly strategy for short-read data using an OLC and a de Bruijn graph assembler is also proposed by Deng et al. (Deng et al., 2015) and shows great promise for metagenome assembly in general.

## Identification of a seed fragment

After assembly it is essential to identify at least one fragment as originating from the target virus. This is most often performed by sequence similarity to known viruses, for example by BLAST analysis (Mokili et al., 2012). In the absence of sequence similarity, contig length, and abundance of the fragment can be an indication for the presence of an abundant organism with a comparably short genome within the metagenome (Bellas et al., 2015). Additional information gained from the sequence alone, like circularity, can also give an indication for the possible viral origin of a fragment (Mokili et al., 2013).

## Linkage of fragments

Even after optimal or nearly-optimal assembly of a metagenome, no full viral target genome might have been recovered, because of drops in coverage or miss-assemblies that prohibit the extension of contigs to full length genomes. Or, in case of viruses with segmented genomes, segments can only be separately assembled. In these cases, additional methods are necessary to link the fragments together. This can be achieved by motif searches, coverage profile binning, kmer profiling, and other methods.

### Motif discovery and search

DNA, RNA, or protein motifs with or without assigned functions are present in the genetic material of viruses (and other life forms). Sequence motifs that are potentially useful for linkage of fragments are motifs that occur several times in a single genome. These motifs can be useful for linkage of missing content, or the linkage of distinct segments. Well-known examples are the conserved motifs that act as mRNA start and poly(A)/stop site in viruses of the order *Mononegavirales (*non-segmented negative-strand RNA viruses) (Kolakofsky et al., 2005; Penno et al., 2015) or motifs that act as leader sequence or transcription-regulation sequences in viruses of the order *Nidovirales* (families *Coronaviridae, Arteriviridae*, and *Roniviridae*) (Pasternak et al., 2006). These elements are conserved in each genome, but the motifs are distinct for the different families. Other positive-strand RNA viruses synthesize subgenomic RNAs with similar or different mechanisms (Miller and Koev, 2000; Lozano and Martínez-Salas, 2015) which can often be linked to the presence of conserved motifs in the genome. The existence of these sequence motifs in *Mononegavirales* and *Nidovirales* could provide an ideal target for motif search and detection strategies for linkage of fragments. Also viruses with segmented genomes, like Influenza A, have been shown to harbor conserved sequence motifs (ElHefnawi et al., 2011).

If the presence of a sequence motif is not obvious, *de novo* motif discovery on a putative seed contig can be performed with motif discovery methods, e.g., with MEME (Bailey et al., 2015), given that this contig is long enough to harbor several occurrences of the motif. Matches of these motifs to other sequences then can be detected with MAST (Bailey et al., 2015). A wide range of applications for motif discovery and searches are available (Das and Dai, 2007) which can accommodate differing data properties and individual preference.

An example of this is the retrieval of a full genome of a red fox fecal rhabdovirus from feces of red foxes from Spain (Bodewes et al., 2014b) that was enabled by detection of a highly conserved junctional motif (Smits et al., 2014). The occurrence of this motif in other, unassigned fragments, was used to link all fragments of the viral genome. The motif was highly specific for the red fox fecal rhabdovirus genome which suggested, given sufficiently long contigs, that motif search could be an effective method to link fragments.

### Coverage profile binning

Binning of coverage profiles refers to the clustering of contigs based on their read coverage, utilizing the differences in abundance of the organism within the metagenome sample. To this end, the read coverage for each contig has to be known, either by extraction of this information from the assembly or by mapping of the reads to the generated contigs. The coverage per base is calculated and contigs with a similar coverage are binned.

Binning of metagenomic contigs by coverage profiles is widely used in general metagenomics (Alneberg et al., 2014) and can be used to recover rare bacterial species from metagenomes (Albertsen et al., 2013). In viral metagenomes, coverage profile binning was used to assemble viral genomes across different human gut metagenomes avoiding the use of reference strains (Nielsen et al., 2014) and to verify cross-assemblies of a novel bacteriophage present in 73% of all publicly available gut metagenomes (Dutilh et al., 2014). However, if amplification is applied during metagenome processing, a coverage bias can be introduced to the sequencing data (Karlsson et al., 2013; Rosseel et al., 2013). In such cases, coverage profile binning does not always have the desired effect of linking fragments from the same source (Smits et al., 2014). Therefore, coverage profile binning can be applied in cases in which amplification is unnecessary or the introduction of a coverage bias was excluded.

### K-mer profiling

Differing sequence composition of the target genome compared to other genomes present in the metagenome can be used to cluster sequences by k-mer frequency profiling. **K-mer profiles** are used in a wide range of sequence similarity searches in bioinformatics. In metagenomics, k-mer frequency profiling is applied for alignment-free similarity analyses between sequences (Sims et al., 2009; Trifonov and Rabadan, 2010; Comin et al., 2015), especially for sequence assembly, quality control (Plaza Onate et al., 2015) and for taxonomic profiling and binning methods (Edwards et al., 2012; Silva et al., 2014; Dröge et al., 2015). There, k-mer analysis allows to overcome data analysis challenges associated with growing data volumes and short read lengths. The sensitivity of taxonomic classification for viral metagenome datasets however does not reach the *de facto* gold standard level of tBLASTX analyses (Vázquez-Castellanos et al., 2014), especially for longer reads(Edwards et al., 2012) and is, like BLAST searches, limited by the quality and comprehensiveness of the reference data set. This is especially true for taxonomic profiling methods, which analyse k-mer content in relation to a set of taxon-specific marker genes or genomes. Binning methods use k-mer profiles to cluster sequences based on similarity of their profiles and allow draft genome recovery (Dröge et al., 2015). Ranking of k-mer profiles, based on their similarity to the seed contig, which is a method similar to binning, was successfully used to link fragments of two rhabdovirus genomes (Smits et al., 2014). Other applications of k-mer analysis in viral metagenomes include nucleotide composition analysis, which are special cases of k-mers (1- and 2-mers) which was successfully used to infer a host for novel picorna-like viral sequences found in gut metagenomes (Kapoor et al., 2010).

KEY CONCEPT 4 **K-mer profiles**

Frequency of all possible DNA sequences of length *k* within one DNA sequence. For example, if *k* = 4, one possible k-mer would be CGTA. In total, a k-mer profile contains frequency information on 4$\hat{\text{k}}$ (256, in case of 4-mers) k-mers.

### Other methods for linkage of fragments

If the sequence similarity of a part of the viral target genomes to sequences in the databases is low, it is sometimes still possible to apply remote homology detection methods (Kuchibhatla et al., 2014), such as PSI-BLAST and HMMER3 (Altschul, 1997; Finn et al., 2011) and profile-profile comparison (HHpred Kuchibhatla et al., 2014), HHblits (Remmert et al., 2012), FFAS (Jaroszewski et al., 2011), WebPRC (Brandt and Heringa, 2009). To apply remote homology detection it is necessary that the viral family of the target genome was identified, for example by the identification of a highly conserved stretch, and that sequence profiles of this families are present in the respective databases [e.g., in pFAM (Finn et al., 2014) or Uniprot (Magrane and Consortium, 2011)] or can be produced from multiple sequence alignments.

Another method to link fragments is to extract all information obtained from an assembly by looking at the original contig graphs (Mulyukov and Pevzner, 2002). Information on adjacency of contigs can be found in such graphs and extracted for genome finishing. This method has successfully been applied when sequencing the gram-negative bacteria *Rickettsia prowazekii* (Nagarajan et al., 2010). Contig adjacency information is especially useful if repeats exist that are longer than the read length, and contigs were split at positions where the reconstruction was ambiguous.

## Finalizing

After linkage of fragments it can be necessary to determine the order of the fragments by generating overlaps between contigs. Order might be inferred from paired end data, if available, but can also be deduced from augmented assemblies, produced by applying different assembly and alignment parameters. Wrong assemblies at contig ends can be the reason that no further overlap has been found by an assembler. Editing of contig ends improves wrong assemblies and facilitates subsequent gap closing with a different assembler.

Combination of different assemblies of the data can help to close potential gaps and confirm the sequences.

## Concluding remarks

In this review, we discussed some methods for assembly and linkage of viral genomes from metagenomes. They overcome often-encountered challenges associated with the extraction of full-length viral genomes from metagenomes: no or little similarity to viral sequences in databases, uneven coverage of the target genomes, and intrapopulation diversity or sequencing errors leading to incomplete contigs.

*In silico* genome finishing methods for recovery of a full-length viral genome from a metagenome are cost-effective and fast because they avoid re-sequencing. Selection of the most effective methods for assembly and finishing of viral genomes depends highly on the sample: host, originating tissue, library preparation, sequencing technique, and depth will lead to different methods for finishing. In general, recovery consists of four different steps (Figure 3): assembly, identification, linkage, and finalizing. All methods are depending on the presence of sufficient sequencing data of the target genome, which in turn is related to a sufficiently high load of the viral target genome in the metagenome sample, and the quality of sample preparation and sequencing protocols in the first place. If target genome loads are not high enough, re-sequencing of the metagenome sample can be a solution, with either a complementary sequencing technique, mate-pair, or paired-end reads, or longer sequences or at a higher depth (Nagarajan et al., 2010; Grard et al., 2012).

In some cases, even though the coverage of the organism is sufficient, viral genomes cannot completely be resolved *in silico* despite exploitation of all possibilities described here. This was the case for the genome of a python nidovirus (PNV, Bodewes et al., 2014a; Smits et al., 2014) which had an unresolvable 5′ end and the PNV genome (accession nr KJ935003) putatively lacks some genome information there. Since also results of 5′RACE PCRs were inconclusive, most likely due to fragmentation of the RNA in the original lung tissue, other options are needed to obtain the complete genome. For instance, another next-generation sequencing platform could be used to confirm the findings of the 454 data. In addition, it could be of interest to set up an *in vitro* culture system to obtain a high titer virus stock with less background and use that as input material, like it was done for the DRV (Osterhaus et al., 1993).

To effectively use k-mer profiles, coverage profile binning and motif search to link fragments, it is essential that long contigs (>1 kb) are produced by the assembly. Coverage and k-mer profile analysis perform better on long fragments because of the noise associated with the high numbers of features in case of k-mers or introduced by uneven coverage (Smits et al., 2014). Motif search is limited by occurrence of the motif, which is more likely in long contigs. Therefore, it is advisable to apply the linkage methods only to well-assembled, long fragments. Successful assembly on the other hand starts with the selection of an assembler suited for sequencing technique and read length, and, if applicable, makes use of paired-end information. By combining assemblies (thus the contigs) or assemblers (thus iterative assembly), the length of contigs can be increased. However, in order to directly recover full-length viral genomes from metagenomes, the development of efficient and dedicated metagenome assemblers that consider the characteristics of viromes and viral genomes is needed.

## Author contributions

RB and AS conceived the study. AS and SS designed the experiments. AS and RB carried out the research. AS and SS prepared the first draft of the manuscript. RB, SS, AR, and WB contributed materials. SS, MK, and AO participated in the discussion and writing of the manuscript. All authors were involved in the revision of the draft manuscript and have agreed to the final content.

## Funding

This work was partially funded by the Virgo Consortium, funded by the Dutch government project number FES0908, by Netherlands Genomics Initiative (NGI) project number 050-060-452 and ZonMW TOP project 91213058. It was also supported by the European Union's Horizon 2020 research and innovation programme under grant agreement No 643476 (COMPARE) and grant agreement No 634650 (VIROGENESIS). It was also supported in part by a grant from the Niedersachsen-Research Network on Neuroinfectiology of the Ministry of Science and Culture of Lower Saxony, Germany. AR holds a Post doc fellowship awarded by the Department of Education, Universities and Research of the Basque Government (Ref. DKR-2012-64) and was partially supported by the Research group on “Systematics, Biogeography, and Population Dynamics” (Basque Government; Ref. IT317-10; GIC10/76).

### Conflict of interest statement

Dr. Albert D. M. E. Osterhaus is partly employed by Viroclinics Biosciences B.V., Rotterdam, Netherlands. The other authors declare that the research was conducted in the absence of any commercial or financial relationships that could be construed as a potential conflict of interest.
